# Narrative Review of the Relationship Between CKD and Diabetic Foot Ulcer

**DOI:** 10.1016/j.ekir.2021.12.018

**Published:** 2021-12-21

**Authors:** Jean-Baptiste Bonnet, Ariane Sultan

**Affiliations:** 1 Transversal Nutrition Unit, Endocrinology-Diabetes-Nutrition Department, University Hospital of Montpellier, Montpellier, France; 2 Mixed Research Unit (UMR) 1302, Institute Desbrest of Epidemiology and Public Health, University of Montpellier, Institut National de la Santé et de la Recherche Médicale, University Hospital of Montpellier, Montpellier, France; 3 PhyMedExp, University of Montpellier, Institut National de la Santé et de la Recherche Médicale U1046, Centre National de la Recherche Scientifique Mixed Research Unit (UMR) 9214, Montpellier, France

**Keywords:** chronic kidney disease, diabetic foot ulcer, epidemiology

## Abstract

Diabetic foot ulcer (DFU) and chronic kidney disease (CKD) are 2 significant complications of diabetes mellitus (DM). Up to 40% of patients with DM are expected to also develop CKD, and 19% to 34% will suffer from DFU during their lifetimes. However, data on the link between podiatric risk and the extent of CKD are scarce. Neuropathy, a key element of the International Working Group on the Diabetic Foot (IWGDF) classification, nevertheless appears to be related to the CKD stage. The incidence of DFU and its poor evolution also appear to be linked to the stage of CKD, with mortality reaching its peak in patients with end-stage renal disease (ESRD). Whatever, the decrease in the rate of diabetic foot amputation observed worldwide, especially for major amputations, is also observed in patients with ESRD. Specific actions taken for patients undergoing dialysis seems to improve the DFU prognosis. CKD and DFU share a number of elements of pathophysiology, the first of which is peripheral arterial disease (PAD). Uremic neuropathy and nutritional status also seem to create a link between the development of the 2 complications. This literature review provides an update on the complex and dynamic relationship between DFU and CKD. It examines the epidemiologic link between CKD and diabetic foot risk, CKD and DFU occurrence, and CKD and DFU prognosis. It focuses on the pathophysiological links between these 2 complications. Finally, it highlights the actions taken to improve management in the ESRD population that have reduced the rate of major amputations in this population by more than half.

DM is a chronic metabolic disease, often complicated by micro- and macrovascular disorders. At the junction of neuropathic and vascular disorders, DFU, usually referred to as “diabetic foot,” stands out as a major complication of DM.^1^ Diabetic foot is a prominent public health issue. In 2013, a study by the French National Institute of Health Monitoring (Institut National de Veille Sanitaire) evaluated the incidence of hospitalizations for foot wounds and amputations at 668 per 100,000, with 25 per 100,000 for people with diabetes.^2^ Furthermore, it is important to note that the incidence of hospitalizations for foot injury of potentially active subjects 50 to 55 years old (600 per 100,000 people) is roughly of the same magnitude, with potential social and functional consequences. Ultimately, 19% to 34% of patients with diabetes will suffer from foot ulcer during their lifetimes,^1^ making diabetes still the leading cause of nontraumatic amputation. Essentially, patients with diabetes have a 12.3-fold risk of undergoing amputation surgery,^3^ which is correlated with a huge mortality rate. According to Larsson *et al.*,^4^ the mortality rate at 1 year and 5 years after amputation is 15% and 69%, respectively. Gök *et al.*^5^ found a 5-year mortality rate of 70% in a study in which age and sex had no significant impact on the mortality rate.

The medical impact of DFU has thus led to the creation of the IWGDF. In 1999, IWGDF guidelines stratified patients with diabetes into 4 groups in relation to the relative risk of DFU incidence.^6^ The classification is as follows: no evidence of neuropathy or peripheral vascular disease (category 0); neuropathy or peripheral vascular disease present but no evidence of foot deformity (category 1); 2 out of the following: foot deformation, lower extremity arteriopathy, or neuropathy (category 2); and history of foot ulcer or lower extremity amputation (category 3). This risk gradation is internationally recognized by health agencies. In addition, the National Institute for Health and Care Excellence has established a similar gradation but with 3 levels: low, intermediate, and high risk. National Institute for Health and Care Excellence’s scoring system has led to the recommendations in the Anglo-Saxon medical world.^7^ Since 2019, the IWGDF has stated that patients with diabetes with ESRD should be classified as category 3 in podiatric risk^8^ even with no history of foot ulcer or lower extremity amputation, on the basis of the observation that patients with diabetes undergoing hemodialysis have a higher risk of developing DFU with a poorer prognosis than other patients with diabetes.^9^ The National Institute for Health and Care Excellence committee had come to the same conclusion in 2015.^7^ Thus, this modification in the IWGDF and National Institute for Health and Care Excellence guidelines highlights a relationship between DFU and CKD. The aim of this work was therefore to review the literature on the link between CKD and DFU. Epidemiology, prognosis, physiopathology, and the link between the IWGDF classification with its components and CKD are discussed.

### CKD and DFU: The Physiopathological Link

It is important to note that diabetes is not the only cause of foot ulcer in patients with renal failure^10^: arterial disease and/or nondiabetic neuropathy can lead to distal ulceration, independently of any diabetes.^11^

#### PAD and CKD

It is not surprising that PAD is a link between ESRD and diabetic foot. In the follow-up phase of the study by Morbach *et al.*,^12^ patients with DFUs had the same long-term survival prognosis if they were on dialysis or had PAD without ESRD. Furthermore, patients with PAD and on dialysis did not have a poorer evolution of DFU than those with only PAD. In this study, dialysis seemed to have had an effect on DFU only through its inflammatory action on the vascular system. This observation is quite similar to those of other studies on patients undergoing dialysis,^13^ even when autonomic neuropathy seems to intervene in the physiopathology of DFU.^14^ In addition, the link between CKD and PAD is well known.^15^ DM is already associated with a higher risk of PAD in the population of patients with an eGFR <30 ml/min per 1.73 m^2^.^16^ Furthermore, an eGFR <30 ml/min per 1.73 m^2^ is a risk factor for atherosclerosis and PAD,^17^ and likewise, a high prevalence of PAD, considered as an ankle-brachial index <0.9, was demonstrated in nondialyzed patients with CKD.^18^ Moreover, the physiological links between oxidative stress and PAD and between oxidative stress and CKD are well known. Yilmaz *et al.*^19^ examined a cohort of patients without diabetes and highlighted this progressive accumulation of asymmetric dimethylarginine (*P* < 0.001), with a possible participation of zinc and selenium deficiency and the known participation of hyperphosphatemia in CKD.^20^ Finally, Margolis *et al.*^21^ pointed to the histologic similarities between mesangial alteration and fibroblasts or podocytes damaged by hyperglycemia. Thus, diabetic nephropathy and PAD are quite the same disease. Nowadays, successful revascularization seems to provide good results in improving the prognosis of DFU patients with ESRD,^22^ and older results need to be reconsidered within the context of today’s improved techniques.

Yet, these observations do not necessarily match with the results of Game’s quite old observation that DFU is temporally associated with dialysis initiation.^23^ Ndip *et al.*^24^ made a similar observation. Is end-stage renal failure accelerated by the inflammatory component of foot ulcer?^25^ Is dialysis treatment initiation the tipping point of renal end-stage failure, through the per-dialytic vascular exploitation on an already precarious vascular background in patients with DM? Indeed, one of the major and immediate effects of dialysis is the intradialytic drop in microvascular blood flow. Intradialytic drop seems to be steeper during dialysis sessions in the diabetic population^26^ versus nondiabetic population.^27^ This issue merits further research.

#### Uremic Neuropathy

One factor that has been explored as a link between DFU and ESRD is neuropathic aggravation through the hyperuremia due to renal failure.^28^ Uremia is known to be an independent cause of neuropathy in ESRD,^29^ and nondiabetic neuropathy seems to be correlated with the level of serum creatinine.^30^ Thus, hyperuremia might worsen preexisting peripheral neurologic disease, affecting wound healing^31^ and promoting infection.^32^ Furthermore, according to the observation of Laaksonen *et al.*,^33^ uremic neuropathy in patients with diabetes does not seem to be improved by dialysis, even though the improvement in patients without diabetes is not a consensus.^34^

#### Nutritional Status and CKD

However, the patient’s overall history should not be overlooked in the management of DFU. Indeed, nutritional status is a central factor in wound healing in general, in DFU,^35^ and in infection for DFU prognosis.^36^ While the link between eGFR and zinc and selenium deficiency has been mentioned,^19^ CKD’s role in altering the levels of vitamins such as vitamin C^37^ and arginine^38^ has been emphasized. Hence, the role of zinc and other trace elements in wound healing should be kept in mind.^39^ In parallel, it is interesting to note that anemia seems to be more prevalent in patients with foot complications and CKD.^40^

#### Global Management

Often forgotten, it is nevertheless important to recall that reaching CKD stage 4 or 5 may be a turning point in the patient’s life. At this point, the nephrologist often becomes the family physician of the patient with diabetes,^41^ and the medical community begins to consider dialysis, vascular access, ESRD dietary plans, and kidney transplant more seriously. Yet, is there still room to talk about foot care? In addition, once a patient enters a dialysis program, treatments occur at a rhythm of 3 sessions per week, which is often exhausting, with increases in anxiety^42^ and their impact on mortality.^43^ As a dialysis program is very time consuming, is there still a place for regular consultations with the podiatrist or the diabetes physician during days without dialysis? Ndip *et al.*^44^ indeed advanced the hypothesis that the foot risk for patients under dialysis might be further aggravated by inadequate or no foot care in dialysis centers, as foot care is often not a medical priority compared with dialysis itself.

### Epidemiology

Up to 40% of patients with diabetes are expected to also develop CKD.^45^ In 2018, according to data from the Registry of the French Epidemiological Network and Nephrology Information Network (Réseau Epidémiologique et Information en Néphrologie), the incidence rate of ESRD treated by dialysis or kidney transplantation was 142 per 100,000 people with diabetes. There are 2 incidence peaks, at 30 to 34 years old and 80 to 84 years.^46^ In 2018, kidney replacement therapy was started for 5,025 patients with diabetes, representing 47% of the newly dialyzed population.^46^ Thus, the review of Narres *et al.*^47^ reported a higher relative risk of ESRD in the diabetic population compared with the nondiabetic population: between 6.2 and 62.0 times higher than in the general population.

### Risk Stratification

Data on the link between podiatric risk and the extent of CKD are scarce.^48^ Recently, Hurley *et al.*^49^ studied diabetic subjects recruited during consultation with a family physician and found a linear correlation between the estimated glomerular filtration rate (eGFR) and an abnormal cutaneous pressure perception assessed with a 10g monofilament, an altered vibration perception threshold, and an abnormal modified Neuropathy Disability Score. They observed a 1% increase in the odds of having an abnormal cutaneous pressure perception for each unit decrease in eGFR. Surprisingly, there was no independent association between a new foot ulceration and a reduced eGFR.^49^ Moreover, with equal glycemic control, the prevalence of diabetic peripheral neuropathy was higher in patients with eGFR <60 ml/min per 1.73 m^2^ than in patients without eGFR <60 ml/min per 1.73 m^2^. This was also the case when the glucose time in range or glucose management indicator measured by continuous glucose monitoring was used.^50^ However, although diabetic neuropathy is one of the key mechanisms in DFU physiopathology, cardio autonomic neuropathy seems to independently predict the progression of diabetic nephropathy and, consequently, of CKD.^14^^,^^51^ If this is the case, some elements of autonomic neuropathy, such as the circadian rhythms of blood pressure disorders,^52^ should be further explored for the global management of CKD and DFU. To our knowledge, there is nevertheless no study highlighting a statistical evidence of a direct correlation between IWGDF podiatric risk and CKD, and the limitations in predictive power seem to be due to the weak relationship between some of the IWGDF components and CKD.

Furthermore, Ndip *et al.*^24^^,^^53^ described podiatric risk repartition in a population under dialysis. Podiatric risk grades 2 and 3 were overrepresented, amounting to 80% to 90% of the population. However, the only robust study regarding the components of the IWGDF risk classification and CKD was the retrospective cohort of Otte *et al.*^54^ A total of 669 patients who had been hospitalized 1,336 times were taken into account (539 with CKD 3, 540 with CKD 4 to 5, and 259 under dialysis treatment).^54^ The study showed no significant differences between CKD stages for peripheral neuropathy or foot deformity but demonstrated differences in history of foot ulceration between the stages (*P* < 0.001).

### DFU Risk

Aragón-Sánchez *et al.*^55^ found a link between albuminuria (with or without impaired renal function) and DFU history. In patients with type 2 diabetes, Wolf *et al.*^56^ demonstrated a positive correlation between creatinine level and DFU risk (*P* < 0.005) in a relatively large population (4,007 patients) and, as for type 1 diabetes, with the gravity of the wound *(P* < 0.001). Furthermore, analysis from patients with type 1 diabetes seemed to suggest a deterioration at 30 ml/min per 1.73 m^2^, but conclusions were difficult to draw with a small population.^56^ ESRD is associated with an increased risk for DFU occurrence, and in the meta-analysis of Kaminski *et al.*,^57^ DM represented a 3.76 (2.21–6.40) relative risk of ulceration in adults with ESRD. The different items of the IWGDF risk scale stood out as risk factors in this meta-analysis: neuropathy, PAD, history of amputation or ulcer, and foot deformity.

In the first prospective study conducted in a population of patients under dialysis, 72% of the population with a DFU was living with diabetes. While diabetes did not emerge as a risk factor in multivariate analysis, neuropathy (hazard ratio = 3.02; *P* = 0.002) and a history of previous foot ulceration (hazard ratio = 2.86; *P* = 0.001) were robust to multivariate analysis. Nail pathology also appeared to be predictive of first ulcer in this dialysis population in multivariate analysis. This may represent a focus for prevention.^58^

### DFU Prognosis

CKD is correlated with a higher amputation rate in patients with DFU.^25^ Several studies have explored the link among ulcer, healing, amputation, and renal function. Despite a small population with type 1 diabetes, Wolf *et al.*^56^ stablished a negative correlation between renal function and foot ulcer, amputation incidence and wound gravity based on the Wagner and Armstrong stages. Similarly, He *et al.*^59^ found that healing prognosis and amputation risk were correlated with renal function stage.

In their review, Jupiter *et al.*^60^ highlighted that CKD was an independent factor correlated with a higher mortality rate in patients with DFU. Some found that CKD and eGFR <60 ml/min per 1.73 m^2^ were correlated with a higher mortality rate for patients with DFU or after amputation.^61^ Van Baal *et al.*^62^ showed a correlation between mortality and an eGFR <30ml/min per 1.73m^2^ in this population, and Carrington *et al.*^63^ showed a correlation between mortality and the serum creatinine level itself. However, is there a linear correlation, or is there a breaking point? It is not possible to answer this question at this point.

In addition, it is known that ESRD is associated with nontraumatic lower limb amputation.^64^ According to Jones *et al.*,^65^ the presence of at least 2 factors: neuropathy, arteriopathy, foot deformity, or nail pathology seems to be correlated with a poor survival index of DFU in populations on hemodialysis, but their study lacked statistical power because of a small cohort. In the meta-analysis of Kaminski *et al.*,^57^ DM represented a 7.5 (5.15–10.87) relative risk of amputation in adults with ESRD on dialysis compared with people without diabetes. Unfortunately, the available data were largely derived from retrospective and cross-sectional studies and, therefore, have limited ability to determine temporal association between risk factors and foot disease. The Dialysis Outcomes and Practice Patterns Study showed a 9-fold greater risk of amputation in the diabetes group versus nondiabetic group,^10^ which is quite similar to the observation in the Medicare ESRD population (odds ratio = 8.93; *P* < 0.0001).^66^

Albuminuria is a known factor of mortality in type 2 diabetes, independently of CKD levels.^67^ Unsurprisingly, it is also linked to the DFU prognosis^55^ and mortality.^68^ More extensive CKD is an independent factor for poor prognosis of DFU in terms of mortality.

CKD and eGFR <60 ml/min per 1.73 m^2^ are correlated with a higher mortality rate for patients with DFU.^69^ In their review, Jupiter *et al.*^60^ highlighted that CKD was an independent factor correlated with a higher mortality rate in patients with DFU. Van Baal *et al.*^62^ showed a correlation between mortality and an eGFR <30ml/min per 1.73 m^2^ in this population. This is concordant with Carrington *et al.*,^63^ who showed a correlation between mortality and the serum creatinine level itself. This effect is stronger than the well-known relation between mortality and ESRD.^12^

### Interventions Aimed at Improving DFU Prognosis

#### A Decrease in Amputation Rates

A decrease in the rate of diabetic foot amputation has been observed worldwide, especially for major amputations,^10^ and this has been also reported for the population under dialysis. Thus, a major analysis of the United States National Registry of dialysis patients^70^ highlighted the significant decline in the global amputation rate between 2000 and 2014. The adjusted rate of all lower extremity amputations decreased from 5.42 per 100 person-years (95% CI, 5.28–5.56) in 2000 to 2.66 per 100 person-years (95% CI, 2.59–2.72) in 2014. This is a relative decrease of 51.0% and applies to both patients with diabetes and those without, although with baseline rates significantly higher for the diabetic group. There was a decrease of 52.8% between 2000 and 2014 in the diabetic group and 48.0% in the nondiabetic group. The amputation rate changed from 8.65 per 100 person-years (95% CI, 8.41–8.88) in 2000 to 4.09 per 100 person-years (95% CI, 3.99–4.19) in 2014 in the diabetic group and from 1.43 per 100 person-years (95% CI, 1.31–1.54) to 0.74 per 100 person-years (95% CI, 0.69–0.79) in the nondiabetic group. Further analysis was completed on this same population from 2000 to 2015 by Harding *et al.*,^70^ who highlighted a 2.8% decrease in minor amputations (toe and foot) per year (from 2.8 to 2.1 per 100 person-years) as well as a 6.4% decrease in major amputations (above knee) per year (from 5.2 to 2.5 per 100 person-years). These observations were the same for men and women and for young (<65 years) and older (≥65 years) patients. According to the data of Margolis *et al.*^21^ for their Philadelphia cohort of patients with diabetes with neuropathic foot ulcers, the global trends between 1991 and 2000 for minor amputations (toe, forefoot, and midfoot) were modified from 2.79 per 100 person-years to 6.01. The data of this study showed that minor amputations reached 72.63% of total amputations.^71^

#### Dialysis Unit and Foot Disease

In accordance with the US cohort, several interventional studies have suggested that specific actions taken for patients undergoing dialysis can improve outcomes.^72^^,^^73^ Thus, different management approaches have been implemented for high-risk populations, especially ethnic subgroups, to improve DFU management.^74^^,^^75^ The study on the Fresenius Dialysis Centers Network in North America should be mentioned in this regard. This work showed that changes in the management of nursing care and the implementation of a foot care program provided by dedicated dialysis nurses were correlated with a 17% decrease in major limb amputations: from 1.30 per 100 person-years to 1.07 (*P* = 0.0034).^73^ As PAD is an irreversible disorder, it is important to maintain a proactive approach to podiatrist care for who underwent a kidney transplant,^76^ especially if the risk of an unfavorable evolution of local infection under transplant immunosuppression is taken into account.

This global improvement in the amputation rate in patients with and without diabetes and the striking effect on major amputations seem to highlight the efforts of dialysis centers to implement a culture of prevention of trophic disorders and to better address vascular handling. However, despite the constant improvement in diabetic foot management, patients under dialysis still have a very poor podiatric prognosis,^77^ and major amputations are still a common outcome. Nevertheless, the dialysis team’s attention to DFU problems and changes in how they are managed might be a key^78^ (Table 1).

**Table 1:** Checklist for a dialysis unit

Dialysis unit organization	• Collaboration with a specialized podiatric clinic • A nurse referent for podiatric issues and monitoring • Multidisciplinary team including a podiatry • Consultation during dialysis sessions • Access to a diabetes team
Each patient	• A referent diabetologist, if applicable • Monthly podiatric examination • Podiatric risk stratification in the medical record • If new ulcer, refer to a specialized podiatric clinic • Adapted shoes if necessary
Foot examination	• personalized therapeutic education • history from the patient in regard to any foot-related issues (ulcer, amputation…) • systematic remove shoes • history of lower limb pain • examination of shoes to check for proper fit and appropriateness, and of the inside of the shoe to identify possible pressure points • appropriate shoes and socks, for example, socks without holes • foot deformities • poor foot hygiene, for example, improperly cut toenails, unwashed feet, superficial fungal infection, or unclean socks • palpation of pedal pulses, both the dorsalis pedis and posterior tibial pulses • foot sensory assessed with a Semmes-Weinstein 10-g monofilament • foot care knowledge and • reminder for a daily foot self-inspection
Diabetes	• HbA1c <8% • continuous glucose monitoring if >3 insulin injections per day • time in range >50% • reduce the risk of hypoglycemia

HBA1c, glycated hemoglobulin.

#### Changes in Demographic Characteristics Change the Podiatric Prognosis Unpredictably

Last, it is important to note that the characteristics of new patients under dialysis are evolving. Indeed, a recent publication based on an American cohort showed a shift in the diabetic population, with a higher proportion of women, Hispanic people, unemployed patients, and patients with higher body mass index, in addition to fewer people with cancer and chronic obstructive pulmonary disease. The impact of these epidemiologic changes on mortality or amputation are therefore quite unpredictable. Mortality and amputation will have to be monitored^71^ because social deprivation, especially among minorities, has been shown to have a huge effect on CKD and DM.

### Conclusion

The incidence of DFU and its poor evolution appear to be proportionally related to the stage of chronic renal failure. However, although the diabetic foot is usually targeted in prevention programs and ESRD is now equivalent to risk stratification category 3 of the IWGDF podiatric risk scale, there is still not enough strong data regarding the relation between CKD and the components of IWGDF podiatric risk. Should patients automatically advance from risk stratification 0 to 3 with the progression of diabetic nephropathy to ESRD despite the absence of history of foot ulcer or amputation? Research on this topic is important, as both chronic ESRD and dialysis are associated with an increased risk of diabetic foot and poor prognosis. This would significantly help in implementing optimized preventive programs.

## Disclosure

All the authors declared no competing interests.
